# The Rapid Development of Glioblastoma: A Report of Two Cases

**DOI:** 10.7759/cureus.26319

**Published:** 2022-06-25

**Authors:** George S Stoyanov, Emran Lyutfi, Radoslav Georgiev, Deyan L Dzhenkov, Ara Kaprelyan

**Affiliations:** 1 General and Clinical Pathology, University Hospital St. Marina, Varna, BGR; 2 General and Clinical Pathology/Forensic Medicine and Deontology, Medical University of Varna, Varna, BGR; 3 Neurology and Neuroscience, Medical University of Varna, Varna, BGR; 4 Radiology and Radiotherapy, Division of Radiology, Medical University of Varna, Varna, BGR

**Keywords:** pathology, case report, tumor growth dynamics, neuroradiology, glioblastoma

## Abstract

Diffuse astrocytic gliomas and their most common and aggressive representation, glioblastoma (GBM), which as per the 2021 World Health Organization (WHO) guidelines is an isocitrate dehydrogenase (IDH) wildtype without alteration in histone 3 and has glomeruloid vascular proliferation, tumor necrosis, telomerase reverse transcriptase (TERT) promoter mutation, epidermal growth factor receptor (EGFR) gene amplification, or +7/−10 chromosome copy-number changes, are fast-growing tumors with a dismal patient prognosis. Herein, we present cases of a 63-year-old male who, despite no evidence of tumor growth, developed a 6-cm tumor, histologically verified as GBM, WHO CNS grade 4, within eight months, and a 74-year-old female in whom a 1.5-cm tumor grew to 43 mm within 28 days, once again histologically confirmed as GBM, WHO CNS grade 4. Other studies using previous WHO guidelines and including up to 106 cases have shown that these tumors have a daily growth rate of 1.4% and can double their size in a period varying from two weeks to 49.6 days. These growth rates further underline the need for extensive surgical resection as disease progression is rapid, with studies reporting that resection of more than 85% of the tumor volume determined on neuroradiology improves survival compared to biopsy or limited resection and resection of more than 98% of the tumor volume statistically improves patient survival.

## Introduction

Glioblastoma (GBM) is a central nervous system (CNS) neoplasm, defined by the 2021 World Health Organization (WHO) guidelines as a malignant tumor with astrocytic differentiation, pronounced cellular atypia (in most cases), and at least one of the following: glomeruloid vascular proliferation, tumor necrosis, telomerase reverse transcriptase (TERT) promoter mutation, epidermal growth factor receptor (EGFR) gene amplification, or +7/−10 chromosome copy-number changes. It is an isocitrate dehydrogenase (IDH) wildtype and has no alteration in histone 3 (H3) [1]. Since the 1930s and 1940s, classical concepts distinguish GBM into two types: primary, which originates de novo, and secondary, which develops from previous lower-grade gliomas (circumscribed as per the WHO CNS tumors guidelines of 2021). The new guidelines and the molecular criteria now classify the classically defined secondary GBM as diffuse astrocytoma, WHO CNS grade 4, IDH mutant [1-5].

These changes have further underlined the aggressiveness of the GBM nosological units as a fast-growing tumor, with no pre-malignant or lower grade precursor lesion, with a dismal patient prognosis [5]. The classical approach for diagnosis of every CNS tumor includes neuroradiological imaging, which in the 21st century has widely become accepted as the surrogate mother of gross neuropathology [6]. Neuroradiology, using both computer tomography (CT) and magnetic resonance imaging, illustrates the tumor's presence well and has been implemented as a screening method in several studies for the early diagnosis of GBM [6]. Although effective for early diagnosis, these studies have proven to be financially ineffective due to the relatively low incidence of primary CNS neoplasms and have shown little to no survival benefit in cases of early diagnosis [7-10]. The only practical implication for these modalities, other than diagnostics of symptomatic patients, remains patient follow-up and the study of tumor growth dynamic and illustrating the de novo nature of GBM origin in cases of previous neuroradiology carried out for other reasons.

Herein, we present the growth dynamics of two cases of GBM developing in a 63-year-old male and a 74-year-old female.

## Case presentation

Case 1

**Figure 1:**
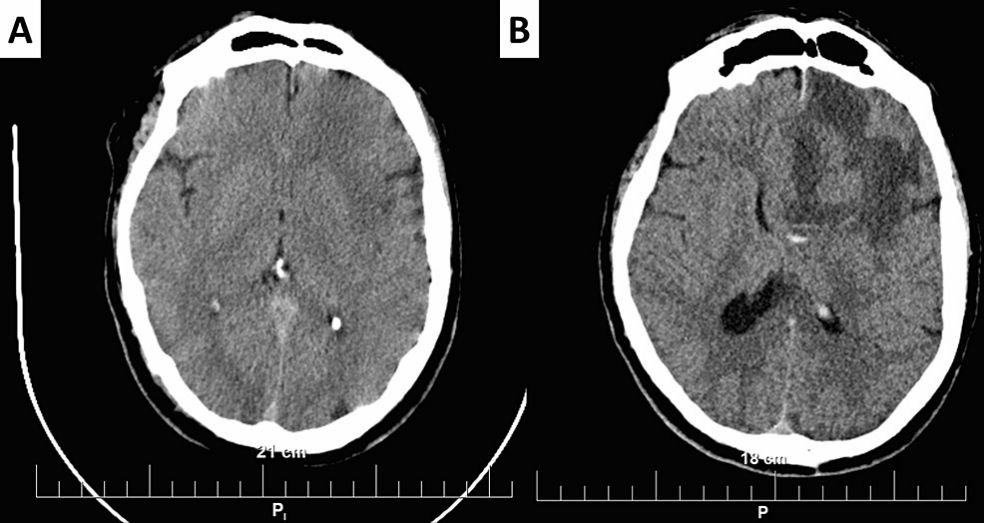
Initial CT (A) and control CT (B) (A) No presence of tumor in the brain parenchyma. (B) Right-sided hypointense tumor with irregular border in the frontal lobe.

The patient, a 63-year-old male, presented to our institution with dizziness and an abnormal gait. Previous medical history included mild hypertension with adequate medication control for the last 14 years. Vitals upon presentation showed body temperature of 37.2°C, oxygen saturation of 99%, blood pressure of 160/80 mmHg, clear vesicular respiration, and soft abdomen. Upon neurological evaluation, right-sided hemiparesis was established, and the patient was sent for CT. The CT revealed no significant vascular findings (Figure 1A). Patient symptoms resolved without intervention. However, the patient was started on antiaggregant therapy and monitored for two days without any new-onset neurological symptoms. Upon discharge, the patient was referred for outpatient neurological follow-up.

Eight months later, the neurologist monitoring the patient referred him for a new cranial CT, as the patient reported severe headaches, mood changes, and visual disturbances, despite good blood pressure control and strict antiaggregant regimens. Control CT revealed a large hypodense lesion in the right frontal lobe, with irregular borders, measuring 6 cm in its greatest diameter, involving both the cortex and the white matter and producing a significant mass effect with central line dislocation of the ventricular system with lateral ventricle dilation (Figure 1B). The patient was admitted to neurosurgery and scheduled for surgery, with the specimens sent for histopathology, confirming GBM, IDH-wildtype, WHO grade 4. The postoperative period was uneventful, O6-methylguanine-DNA methyltransferase (MGMT) analysis revealed promoter methylation, and despite radio and chemotherapy with temozolomide, the patient expired 172 days after surgery due to disease progression.

Case 2

**Figure 2:**
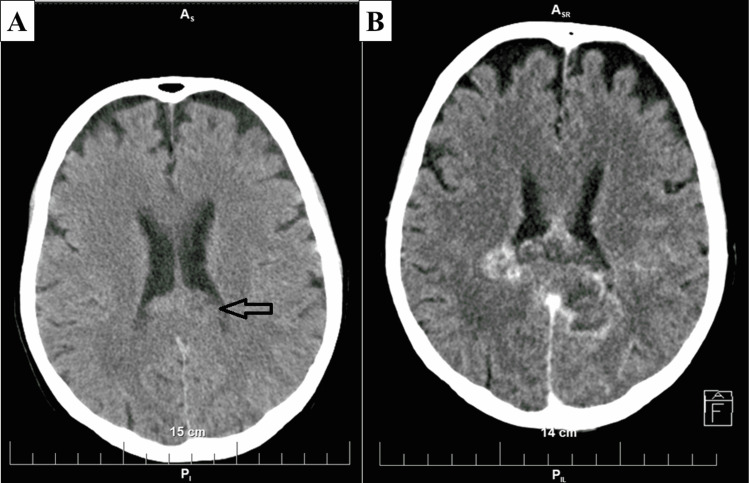
Initial CT (A) and control CT (B) (A) Isointense lesion in the splenium of the corpus callosum (arrow). (B) Bilateral tumor with irregular borders, with a significant increase in size.

The patient, a 74-year-old female, presented to our institution with a one-week history of visual disturbances, new-onset dizziness, and temporary loss of consciousness. Previous medical history included cervical spondylosis. Vitals upon presentation showed the patient was afebrile, with oxygen saturation of 98%, blood pressure of 150/100 mmHg, clear vesicular respiration, and soft abdomen. Upon neurological evaluation, right-sided lower limb monoparesis was established, and the patient was sent for CT, which showed an isointense lesion, 1.5 cm in its greatest diameter in the splenium of the corpus callosum (Figure 2A). Due to the characteristics and the size of the lesion and the lack of new-onset or symptom progression, the patient was referred to outpatient neurological follow-up with control CT after a month for reevaluation for neurosurgical intervention.

After 28 days, the patient was referred by her outpatient neurologist for control CT due to a mild progression of visual symptoms. The control CT revealed significant disease progression with the lesion now being with a hypointense central zone, irregular borders, involvement of both cerebral hemisphere occipital lobes, producing a mass effect with dislocation of the lateral ventricles and internal hydrocephalus, with size in its greatest diameter measuring up to 43 mm (Figure 2B). The patient was admitted to neurosurgery and scheduled for surgery, with the specimens sent for histopathology, confirming GBM, IDH-wildtype, WHO grade 4. Despite the early postoperative period being uneventful, the patient's neurological status deteriorated rapidly. MGMT analysis revealed no promoter methylation, and the patient expired 27 days after surgery, before the initiation of treatment due to further disease progression.

## Discussion

Due to the changes in the WHO CNS tumor classification, first introduced in 2016 with IDH status requirements and further tumor type subdivision in the 2021 criteria with H3 status and genetic markers, few studies have studied the growth dynamics of GBM as per the new guidelines [1]. Based on older classifications, previous studies have underlined the rapid growth of diffuse gliomas and especially GBM [1]. One such study carried out by Stensjøen et al. found that the daily rate of tumor volume increase was 1.4%, and the time to tumor volume doubling was 49.6 days, found by repeated neuroradiological studies in 106 patients before surgery [11]. However, the study is based on the previous classification, including slower-growing forms of IDH mutants (now astrocytoma, IDH mutant, WHO CNS grade 4). If we assume that 10% of the tumors in the depicted sample lose their classification as GBM, as per epidemiological data on IDH mutant and wildtype forms, then GBM, according to the WHO criteria from 2021, warrants an even more aggressive growth rate. In a smaller group (n = 32) with similar characteristics and using similar methods, Wang et al. found that the time to the tumor volume doubling was 17 days [12]. In a case report of a 60-year-old man, Zhang et al. illustrated neuroradiological progression of GBM from a 7 mm to a 13 mm lesion by day 12, 17 mm by day 23, and involvement of almost the entire hemisphere seven months after the initial CT, suggesting GBM doubles in size in about 10 days [13]. Our cases further underline and support the reported data, especially in the second case, where the tumor volume doubled in less than a month.

Given the rapid growth of the tumor and its diffuse nature, which cannot be fully established neuroradiologically alone, neurosurgical interventions in GBM are not only difficult methodologically and with resulting neurological deficits but are key to patient survival. In an analysis of the survival of more than 400 patients according to the old classifications, Lacroix et al. found that only excision of more than 85% of the neuroradiologically observed tumor volume showed an improvement in patient survival compared to only biopsy or limited resection, with a statistically significant difference in survival being observed when the resection included more than 98% of the neuroradiologically established tumor volume [14]. The role of radical resection was first underlined by one of the pioneers of modern neurosurgery, Walter Dandy, who reported five cases of hemispheric resection, four in patients with gliomas, where despite high postoperative mortality (one patient died 48 hours after surgery due to bleeding and another after two weeks due to pneumonia), three patients survived for a prolonged period varying between three months (in a patient with a butterfly glioma) and three and a half years after surgery [15].

## Conclusions

Despite the taxonomy changes and the new requirements in the WHO 2021 CNS tumor classification for diffuse astrocytic gliomas, GBM remains the most common and aggressive CNS tumor in adults. Multiple previous studies, including nosological units no longer classified as GBM, have underlined the aggressive growth pattern, where the tumor volume doubles for a period between two weeks and 49.6 days on average. These studies include cases of tumors that are now known to be genetically less aggressive and with a slower growth rate. If applied to the 2021 guidelines, then GBM has an even faster growth rate, probably one of the highest in human pathology. As seen in the cases reported by us, a 6-cm tumor engulfing nearly a whole lobe can develop de novo in less than eight months, and a tumor can more than double its size in a single month.

## References

[1] Louis DN, Perry A, Wesseling P (2021). The 2021 WHO classification of tumors of the central nervous system: a summary. Neuro Oncol.

[2] Scherer HJ (1940). Cerebral astrocytomas and their derivatives. Am J Cancer.

[3] Scherer HJ (1938). Structural development in gliomas. Am J Cancer.

[4] Kayabolen A, Yilmaz E, Bagci-Onder T (2021). IDH mutations in glioma: double-edged sword in clinical applications?. Biomedicines.

[5] Zepecki JP, Snyder KM, Moreno MM (2019). Regulation of human glioma cell migration, tumor growth, and stemness gene expression using a Lck targeted inhibitor. Oncogene.

[6] Perry A Perry A,  Brat DJ  Brat DJ (2017). Practical Surgical Neuropathology: A Diagnostic Approach. https://www.sciencedirect.com/book/9780323449410/practical-surgical-neuropathology-a-diagnostic-approach.

[7] Jakola AS, Myrmel KS, Kloster R, Torp SH, Lindal S, Unsgård G, Solheim O (2012). Comparison of a strategy favoring early surgical resection vs a strategy favoring watchful waiting in low-grade gliomas. JAMA.

[8] Komotar RJ, Starke RM, Connolly ES (2008). Brain magnetic resonance imaging scans for asymptomatic patients: role in medical screening. Mayo Clin Proc.

[9] Neugut AI, Sackstein P, Hillyer GC, Jacobson JS, Bruce J, Lassman AB, Stieg PA (2019). Magnetic resonance imaging-based screening for asymptomatic brain tumors: a review. Oncologist.

[10] Verduin M, Compter I, Steijvers D (2018). Noninvasive glioblastoma testing: multimodal approach to monitoring and predicting treatment response. Dis Markers.

[11] Stensjøen AL, Solheim O, Kvistad KA, Håberg AK, Salvesen Ø, Berntsen EM (2015). Growth dynamics of untreated glioblastomas in vivo. Neuro Oncol.

[12] Wang CH, Rockhill JK, Mrugala M (2009). Prognostic significance of growth kinetics in newly diagnosed glioblastomas revealed by combining serial imaging with a novel biomathematical model. Cancer Res.

[13] Zhang YY, Ruan LX, Zhang S (2016). Rapid progression of glioblastoma multiforme: a case report. Oncol Lett.

[14] Lacroix M, Abi-Said D, Fourney DR (2001). A multivariate analysis of 416 patients with glioblastoma multiforme: prognosis, extent of resection, and survival. J Neurosurg.

[15] Dandy WE (1928). Removal of right cerebral hemisphere for certain tumors with hemiplegia: preliminary report. J Am Med Assoc.

